# Being a bridge: Swedish antenatal care midwives’ encounters with Somali-born women and questions of violence; a qualitative study

**DOI:** 10.1186/s12884-015-0429-z

**Published:** 2015-01-16

**Authors:** Ulrika Byrskog, Pia Olsson, Birgitta Essén, Marie-Klingberg Allvin

**Affiliations:** School of Education, Health and Social Studies, Dalarna University, S-791 88 Falun, Sweden; Department of Women’s and Children’s Health, Uppsala University, Akademiska sjukhuset, S-751 85 Uppsala, Sweden; Centre for Clinical Research, Nissers väg 3, S-791 82 Falun, Sweden

**Keywords:** Violence, Somali born women, Antenatal care midwife, Communication, Trustful relationships, Networking, Person-centered, Qualitative method

## Abstract

**Background:**

Violence against women is associated with serious health problems, including adverse maternal and child health. Antenatal care (ANC) midwives are increasingly expected to implement the routine of identifying exposure to violence. An increase of Somali born refugee women in Sweden, their reported adverse childbearing health and possible links to violence pose a challenge to the Swedish maternity health care system. Thus, the aim was to explore ways ANC midwives in Sweden work with Somali born women and the questions of exposure to violence.

**Methods:**

Qualitative individual interviews with 17 midwives working with Somali-born women in nine ANC clinics in Sweden were analyzed using thematic analysis.

**Results:**

The midwives strived to focus on the individual woman beyond ethnicity and cultural differences. In relation to the Somali born women, they navigated between different definitions of violence, ways of handling adversities in life and social contexts, guided by experience based knowledge and collegial support. Seldom was ongoing violence encountered. The Somali-born women’s’ strengths and contentment were highlighted, however, language skills were considered central for a Somali-born woman’s access to rights and support in the Swedish society. Shared language, trustful relationships, patience, and networking were important aspects in the work with violence among Somali-born women.

**Conclusion:**

Focus on the individual woman and skills in inter-cultural communication increases possibilities of overcoming social distances. This enhances midwives’ ability to identify Somali born woman’s resources and needs regarding violence disclosure and support. Although routine use of professional interpretation is implemented, it might not fully provide nuances and social safety needed for violence disclosure. Thus, patience and trusting relationships are fundamental in work with violence among Somali born women. In collaboration with social networks and other health care and social work professions, the midwife can be a bridge and contribute to increased awareness of rights and support for Somali-born women in a new society.

## Background

Violence against women (VAW) is a multifaceted global public health problem and recognized as a violation against human rights. There are increasing recommendations to include routine questions about exposure to violence during health care visits, both in Sweden and internationally [1]. The midwife in antenatal care (ANC) is a key person, due to repeated encounters from early pregnancy to postpartum with women and their partners [2,3].

The motivation for including routine questions about violence in health care visits is based on scientific evidence indicating that violence against women is associated with serious health problems, such as adverse maternal and child health [4,5]. Increased levels of gynecological complaints, elective abortions, and psychological distress are reported among women subjected to intimate partner violence (IPV) or sexual violence [6-8]. Furthermore, increased pregnancy related symptoms, risk for delivery complications, instrumental deliveries [9,10], and increased antenatal hospitalization [4] are reported. Additionally, more perinatal deaths, low birth weight (LBW), and small for gestational ages (SGA) infants are born to women exposed to violence [5,11]. A long-term effect among the children is increased risks for psychological ill health later in life, due to disturbed early attachment between the child and a violence or trauma exposed mother [12]. Violence is associated with stigma worldwide and through asking questions within the health care system, society makes the public health problem visible, signals that violence is unacceptable, and that support can be offered [13].

The World Health Organization (WHO) classify violence as deprivation, psychological, physical, and sexual violence at self-directed, inter-personal (including IPV), and collective levels [14]. In Sweden, foreign born women have an increased risk of interpersonal violence exposure and of mortality directly linked to violence compared to Swedish born women [15]. There is an increased risk of IPV during the first year after delivery if the woman or her partner is born in a country outside Europe [16]. Refugee women from low income and/or conflict settings are more likely to have a history of exposure to violence and experiences of trauma during flight and displacement [17,18]. Exposure to IPV may increase both during flight and post migration, due to changed family power dynamics, uncertain asylum processes, economical constraints, and traumatic experiences [19-21].

In Sweden, antenatal care (ANC) is offered free of charge to all pregnant women. The uptake is 99% and the midwife as the main provider independently provides ANC for the woman during the 10-12 visits of a normal pregnancy [2]. The ANC midwife is close to the pregnant woman and her partner, and encounters patients from all parts of society, including immigrants. Somali-born women coming from a war affected country constitute one of the largest groups of birth-giving immigrant women in Sweden [22]. Increased perinatal deaths, increased risk for emergency caesarian sections, maternal ill health during pregnancy, and late access to maternity health care services are reported among childbearing Somali born women after migration compared to Swedish born women [23-25]. Adverse maternal and perinatal health and outcomes are reported also in other high-income settings receiving Somali refugees [26]. A pre- and post-migration maternal effect based on socio-economical [27] and cultural influences in which strategies of Somali refugee women interfere with a contrasting western bio-medical maternity health care system [28] might contribute to late access and suboptimal outcomes. In addition, language barriers [29-31] and difficulties navigating a new maternity health care system [32] are complicating factors.

The increase in the number of Somali born women in Sweden, their reported adverse childbearing health after migration [23-25,33], and possible links to violence pose a challenge to the Swedish maternity health care system. The midwife is situated at the intersection between the Somali born woman and the health care system and is increasingly expected to implement the routine of identifying violence exposure. However, there are a lack of studies exploring the encounters between Somali born women and midwife in relation to violence. Thus, the aim in this study was to explore ways ANC midwives in Sweden work with Somali born women and the questions of exposure to violence.

## Methods

A qualitative approach of individual qualitative interviews [34] with thematic analysis [35] was applied in order to capture the experiences and perceptions of the midwives. The data collection and reporting adheres to the RATS guidelines for reporting qualitative studies [36].

### Setting and participants

A purposive sample of midwives (n = 17) was recruited from 11 ANC clinics; one to two midwives from each clinic, in the middle and north of Sweden. Before recruitment, the first author (UB) informed superintendents or midwives at the actual clinics about the study by telephone and by e-mail. Inclusion criteria for the study were being a registered midwife, more than two years of work experiences at the actual clinic, and working with a multi-cultural mix of patients, including Somali born women. In order to achieve a variety of settings, ANC clinics in both large metropolitan areas and smaller cities were included and the working experiences of the midwives varied between 5 and 20 years. One participant was included in the study despite only being in the position for 1 ½ years, as her function as a midwife for asylum seeking- and refugee women only provided additional perspectives to the study. Three approached clinics refrained from participation due to heavy workload. Before inclusion in the study, each informant was given oral and written information about content, confidentiality, and the voluntary nature of participation and oral and written consent for participation was obtained. Ethical approval was obtained from the Regional Ethical Review Board of Uppsala, Sweden (2008/226).

### Data collection

The present study was part of a larger data collection also including Somali born women’s perspectives [37]. Data were collected in 2012 and 2014. The first author (UB) conducted the interviews at the participants’ workplaces. The interviews, lasting between 30 to 90 minutes, were recorded digitally after permission from the informants. Initially, informants were asked to recall a situation they had encountered that included a Somali woman and an aspect of violence. Follow-up questions were open ended and based on a topic guide, including reflections on Somali women and different forms of violence, the midwifery role in relation to Somali born patients and violence, barriers and facilitators in counseling violence and well-being with Somali born patients, and the midwife’s role in counseling about violence in general. In interviews where the informant could not recall a situation to share, interviews started with the questions based on the topic guide.

### Data analysis

Data consisted of 14 hours of recorded interviews that were transcribed verbatim. To obtain an overview of the data, the recordings and transcripts were listened to and read several times. Thematic analysis, according to Braun and Clarke [35], was chosen for the analysis due to its’ inductive systematic yet flexible character, and the possibility to achieve ‘thick descriptions’ of data. The data was approached inductively and extracts consisting of raw data concerning the role of midwifery in relation to violence, the encounters with Somali born women, and violence were identified. From these extracts, initial codes close to raw data were identified and grouped on a semantic level by the first author (UB). Thereafter, temporary subthemes were constructed and crosschecked against groups, initial codes, and the transcripts. The subthemes were finally arranged into three themes with one overarching latent theme. Groups, subthemes, and themes were repeatedly discussed and revised by all authors during the process of analysis.

## Results

Three themes with subthemes were identified which illustrated different aspects of the midwives’ encounters with Somali born women when approaching, identifying, offering support and counseling about violence (Table 1). An overarching crosscutting latent theme was that the midwife constituted a bridge between the Somali born woman and the new society.

**Table 1:** **Themes and sub-themes of approaching, identifying, and counseling violence in midwifery-encounters with Somali born women**

**Themes**	**Subthemes**
Approaching violence in the care encounter	*See each woman’s uniqueness*
	*Establishing trusting relationships*
	*Achieving cultural competence*
Identifying violence across diversity	*Facing different views and spectra of violence*
	*Finding suitable ways of asking*
	*Shared language is a key*
Support and counseling in ‘the new society’	*A bridge to support and awareness of rights*
	*Encounter the ‘here-and-now-approach’*

### Approaching violence in the care encounter

The first theme highlighted three components of importance for the midwives when approaching the issue of violence in their care encounter with Somali born women. Each woman’s uniqueness, trustful relationships and cultural competence were central.

#### See each woman’s uniqueness

To see the individual woman was described as a crucial element in the role of the midwife. This meant going beyond ethnicity and socio-demographic background in the encounter with a woman, and avoiding generalizations regarding both violence and other aspects of health.

*“I mean, my goal is to care for every woman, as the woman she is, regardless of her origin or religion.” MW13*

Nuance was important for the midwives when they shared experiences of encounters with patients of different nationalities and they refrained from describing features associated with violence and immigrant women as something connected to a particular ‘group’. Nevertheless, an act of balance was depicted in the encounters with immigrant women, in which the midwives had to navigate between their own internalized values and the woman’s way of living or acting, across language barriers. With the use of empathy, and dialogues with colleagues, distances based on divergent life situations were addressed, but the balance could still be delicate to handle. This was further depicted in situations when a Somali born woman’s response differed from the ‘mainstream’ response or was difficult to interpret. Therefore, there was a tendency to present ‘cultural’ explanations on group level, which was fuelled by colleagues’ stories, despite the wish to be person-centered. Furthermore, informants described how group-level constructions were sometimes reinforced by the Somali women themselves.

*“Often I think that, yes, they think similar to us.… And then suddenly you walk into like a wall. Aha, ok: (the patient says:) ‘the culture tells us that we are not allowed to think about this that has happened in the past…’ MW11*

#### Establishing trustful relationships

Approaching violence was considered a process. Violence exposure might not be revealed at the first occasion, but on later occasion, when the woman was ready, then she would know where to turn for advice and support.

*”…’even if I don’t tell it the first time, then I know, that if I go to the ANC clinic … the question will be asked again. And this time I might dare to tell and know where to turn to after’.” MW13*

Trust was essential in the work with violence-related issues. A good reputation of midwifery in the women’s networks, an understanding of the midwife’s confidentiality, and a feeling of being listened to and seen by the midwife were considered important for establishing trust. Informants felt the midwifery profession was highly valued by Somali women and was a good foundation for building a trustful relationship. Conversations about violence could actually promote a deeper interaction and closeness between the midwife and the woman. Finding ways to connect to the individual woman, such as sharing some words in Somali, could deepen the relationship and the trust building process. However, sometimes time throughout several pregnancies were required before a woman opened up and revealed her situation.

*“Some Somali women are also more difficult to come near, to open up in the beginning, instead you have to build that trust up … before they dare…”MW4*

#### Achieving cultural competence

Clinical experiences, including the skill ‘to read the woman’, provided a base in the encounters with Somali born women in relation to violence. This was by some midwives considered as a sufficient foundation of knowledge and tools for encountering the issue of violence with Somali born women. To consult more experienced colleagues was recommended to new colleagues if they were uncertain regarding how to approach Somali born women with questions about violence. However, the importance of finding a personal strategy with each patient and allowing time for this process were stressed.

Informants felt they had received satisfying knowledge and guidelines concerning violence in general terms when the routine questions of violence had been introduced and with time, raising the questions of violence with different women became a normal part of the work. However, some midwives experienced that in relation to the Somali born women, they lacked the background knowledge regarding cultural and religious conceptions of health, family life, value systems, and violence. They described how the responsibilities for achieving this type of knowledge were solely on their own shoulders. In one clinic, the management’s priorities regarding education had been reflected on and compared with the education regarding homo, bi and transgender persons, but the informant had felt it was a controversial issue to raise:

*“For we said, we meet so many Somali women and from other cultures, but most Somali. And we know so little about their culture. Because out of the pregnant we have…we admit about 450 yearly and out of them, maybe five are same-sex couples. But how many more Somali women…” MW17*

### Identify violence across diversity

The second theme described the midwives’ strategies for facilitating questions to identify violence. In relation to Somali born women, particular challenges were encountering different views on, and forms of, violence while facing language barriers.

#### Facing different views and spectra of violence

The midwives described how sometimes they found it difficult to define and discuss violence in the care encounter, particularly psychological and economical violence, due to possible divergent roles and relations within families. One area of uncertainty was whether their Somali born patients might consider non-consensual sex within marriage as acceptable or as sexual violence.

*”…there the difference comes again. How do they view this with marriage and sexual obligations, if you should call it like that? Are you allowed to say no within marriage or not?” MW1*

Female genital mutilation/cutting (FGM/C) was another example the Swedish midwives and Swedish society considered a kind of violence, but rarely mentioned as violence by the Somali born women. The women themselves seldom initiated the topic of FGM/C during care encounters. Nevertheless direct questions from the midwife were answered and the general opinion among the midwives was that the women appreciated this and that FGM/C should be raised during the ANC encounter. Midwives remembered women who had shared traumatic memories from the act, but the midwives mainly raised the view that FGM/C was perceived as a natural tradition for a Somali woman. This did not necessarily mean the Somali born women would perform FGM/C on their own daughters – rather the opposite opinion had been noted.

*“But you never get an answer on violence exposure with regard to that they are circumcised. Never. It does not count as violence, even if they have been held down by several adults.”MW9*

Thus, the midwives stated all individual’s perspectives on violence were influenced by prevailing norm systems, own experience, and by ongoing public debates. ‘Normal’ behavior opposed to what was considered as violence could differ depending on backgrounds and this was made visible in the encounters with Somali born women.

Although the existence and content of guidelines regarding the focus for violence questions varied in different clinics, the main domain was IPV. The Somali born women brought a broader spectrum of violence and experiences from a war torn reality into the care situation, which had led informants to question which violence they should actually ask for. Some midwives described how their knowledge about the women’s different situations broadened their questions in relation to violence and that this could improve the care they provided.

*”…’my father and my brother they died and they were shot’…maybe it is not exactly that you want to grasp when you ask this violence question. But, I think anyway that it is interesting to get a hold on what they carry in their baggage … And if you then have witnessed close relatives being killed. And how life can be changed in only one minute. It can actually impact how you feel about life and to give birth, I believe.” MW9*

The midwives seldom associated Somali born women with IPV. One explanation was their uniform descriptions of Somali women’s characteristics, which were based on their own perceptions and opinions shared by colleagues. Although vulnerability was described, the Somali women were predominantly described as strong and independent.

*”…Somali women, they have a lot of their own will. And I believe that…yes, they kick the man out when they don’t want him anymore. But then he is allowed to come back and visit sometimes” MW3*

Many Somali born women appeared to live alone and were only periodically with the partner, due to war-related reasons and divergent family patterns. It was thought that this might reduce the risk of IPV exposure – and thus also the incitement for asking about IPV to these women could be lower.

It was perceived that most IPV was revealed after the acute situation had already passed, irrespective of the women’s backgrounds. One possible reason for the Somali woman remaining silent could be the fear of a connection between the ANC clinic and social authorities.

#### Finding suitable ways of asking

Asking women questions about violence exposure had been introduced as a routine in all clinics but one, and almost all informants considered it a midwifery task. The motivation behind asking was the concerns for the individual woman and the unborn child. The risks of IPV escalating during the pregnancy and the woman’s’ emotional process during pregnancy were also mentioned.

Questions of violence were often imbedded in admittance-routines at the ANC clinics, if the partner was not present. If the partner was present, it was crucial to remember to ask later during the pregnancy. This was seldom a problem with the Somali women, as they often came to the midwifery visits without the partner. As Somali born women sometimes started ANC visits later during pregnancy, there was a risk of overlooking the violence questions due to having less time with the individual woman. One strategy for catching those who were admitted late was the routine of asking both early and late during pregnancy; however, the second time was sometimes forgotten or avoided due to a feeling of being too focused on violence in the encounter with the woman. It was stated a midwife is obliged to perform many tasks during a woman’s visit. Language interpretation could obstruct the midwifery visits, and while many midwives tried to keep this part short and concise, others considered it possible to prioritize and add extra time to visits when interpreters were present or when patients came from war-affected countries.

Strategies for asking about violence included having clear and direct violence questions included in the admittance routine. However, sometimes a less straightforward approach was used, as it might frighten particularly refugee women if the midwife was too direct:

*“I don’t express ‘violence’, I rather ask, ‘is your husband kind with you?’ And maybe they look at me: ‘ yes, well, he is kind…’ kind is maybe a strange word too. But, you don’t know how to start. … You know, you want to go around. It is difficult to be very straightforward. But sometimes you must be very explicit and say ‘hit’.” MW15*

Having come from a war torn area was considered a natural base to start a conversation on violence, as the questions then might be less shameful for the Somali born women.

*“But maybe for Somali women, I used to say, that you come from a country where there is much violence and have you been subjected to anything…If it might be very shameful, it is easier to approach it, I mean: well, you come from a country…like that. Because…then the shame is not on them in a way.” MW12*

However, based on the urge to move away from ethnic stereotypes, opposing strategies were also described.

*”But this we have also discussed, that they should not believe that we ask them because they are foreign born. No, so therefore I am, as with the Swedish women, very distinct with that this we ask everyone. It’s not that ‘I suspect you are being hit and how does it look like over there’ and so on.” MW11*

#### Shared language is a key

Shared language was considered an important key for the Somali born woman to be able to understand her rights in a new society and as a key in the midwives’ encounter with a woman when trying to identify violence exposure. The midwives realized women were less likely to talk about a violent life situation with a third party involved, due to the risk of gossip, and were restrictive with the use of friends or family as language interpreters. Even so, women could be afraid of gossip despite talking through an interpreter with professional confidentiality.

Although some midwives preferred well-known interpreters on site who were used to the conversations regarding violence, many clinics used interpreters over telephone to avoid an interpreter from the women’s network. However, the telephone itself could be a limitation due to the unnatural situation it creates in the care encounter. Written information often complemented the oral conversations regarding violence, which further added to the limitations in relation to the Somali born women. Even if information was provided in different languages, midwives were sometimes unsure of the women’s literacy.

*“Language makes a big difference. And that it might be harder to embrace information. This with reading, writing, who to contact, who can help me if I need to leave here. That’s the big difference. Language is a big problem. … We like to give out brochures and papers and so on.” MW2*

A relationship and dialogue with a woman with limited Swedish skills was more likely to remain on a surface-level with less room for individuality and deeper reflections than with a patient fluent in Swedish.

*“I notice that when I talk and use an interpreter, I simplify the Swedish language a little and when I talk with the women without an interpreter, who understand poorly but still have a little Swedish. Then it happens that you give short commands and speak simply so they will understand.” MW3*

Thus, the risk of losing nuances through simplified language could complicate the process of communicating about violence with Somali born women. Furthermore, being dependent on a professional interpreter’s values without control over the conversation was described as a limitation.

### Support and counseling in ‘the New Society’

The midwives’ roles were to raise awareness and visualize violence exposure, and when needed, guide the woman to support and counseling. Time to listen without conveying stress was mandatory when asking the question. If ongoing violence was revealed, it was crucial to prioritize this, even if it required giving up private time. The third theme highlighted the midwives’ experiences and reflections regarding different aspects of support and counseling in relation to the Somali born women they encountered.

#### A bridge to support and awareness of rights

All clinics had functioning structures for follow-up and support for women with ongoing or earlier violence exposure. The same support and referrals could be offered to anyone, regardless of nationality, but the individual woman’s willingness or readiness to receive support could differ.

An immigrant woman could have less knowledge about support systems and women’s rights in the new society. Hence, questions of violence facilitated opportunities for informing about rights and available societal resources.

*“There are good things in it for the woman. And specifically that, that they would know their rights, especially the foreign girls” MW1*

At some units, particularly at family health centers, midwives worked in networks with social workers, health care professionals, and bi-lingual health advisors of Somali origin. This created a more holistic approach in the midwives’ encounter with Somali born women and they could pay attention to the whole life situation for the woman. The concept of collaboration across professions provided space for parent group education with shared leadership, depending on the topics. Besides increasing knowledge about pregnancy and delivery, parent group education could be an arena for discussing men’s and women’s roles and existing laws and rights linked to the topic of violence. The parental groups were also a forum for lonely Somali born women for building social networks.

*”…there I think, if we could get all our Somali women to step into our groups. I think we would do a lot of good there…because you should probably dare, you get a bigger opportunity to come into the Swedish society and know how it works. What laws we have here. And learn to know, that ‘this was not ok, this that I have been subjected to earlier. You don’t act in that way’.” MW13*

However, parent education groups for Somali born women often required several trials, and time and patience. Some clinics had stopped trying due to economic constraints or to a big dropout of the Somali born participants. Instead some informants described how they on invitation visited women’s groups organized by Somali networks or other actors in the community.

#### Encounter the ‘Here-and-Now-Approach’

In their encounters with Somali born women, the midwives had gained insight into alternative strategies for dealing with the consequences of violence and difficulties in life. Somali women who with a positive mind or without complaint had carried and given birth to babies conceived out of rape had impressed:

*“And I asked her how she feels concerning the baby… ‘Yes, but the baby had no guilt in this’. Very interesting, really. That she was expecting the baby and happy for the coming baby. Because it was not to blame. And it is also very great to think like that”. MW1*

Somali born women’s readiness to share violent experiences, emotional load, or to receive counseling was depicted differently among the midwives. Predominantly, it was perceived that many Somali born women applied the strategy of leaving past traumatic and violent events behind and chose to concentrate on life here and now. This assumption was supported by midwives’ perception of a low use of psychological counseling among the Somali born women, despite histories of their own or relatives’ exposure to war-related violence and family separations. A culture-specific contentment in life, social networks, strong integrity, shame or unfamiliarity from the pre-migration context were suggested as explanations for a perceived low need for sharing more than the most necessary things about the past in the care encounter. It was suggested that instead psychosomatic reactions, such as sleeping difficulties or pain, might be increased among the Somali born women; however, this was not always easy to distinguish from common pregnancy complaints.

Whether the ‘here-and-now-approach’ was a well-functioning long-term strategy or not and how deep the midwife should engage themselves in the women’s experiences from the past were reflected on. Motivated by respect for boundaries set by the individual woman, some midwives could be content with themselves offering support, whether it was received or not. However, the consequences of not sharing experiences or receiving professional counseling were a concern for others, which led some midwives being prone to overcome initial distances in the encounter.

*“…it has to come out somehow at some point, I think. I mean, of course if you have seen your dad being killed and yourself been subjected to rape, like someone told last week and then she says; ‘I have not told this before, because I don’t want to talk about it’. Of course, in some way it must… MW12*

When a woman appeared distressed, some midwives used the strategy of offering more frequent visits to the clinic. Thus, they acted as bridges when there was hesitance regarding professional psychological support. Trying to determine whether the woman was surrounded by a social network was another strategy. When the response from the patients regarding violence exposure or the need of support was low, a perceived natural sisterhood among Somali born woman was a comfort.

*“I perceive the Somali women overall as incredibly strong, secure and content. And you seldom need to go in and help them. They manage very much by themselves. I believe many live in a sisterhood. That they have many relations and many friends. And they talk a lot with each other. I think.” MW10*

However, it was difficult to know if the informal support system was sufficient and whether hardships such as violence were shared or not. A special concern was newly arrived Somali born women who appeared lonely, vulnerable, and dependent on the decisions made by husbands or extended families.

*“And that they are very lonely we have discovered now when we have run our parenting groups. You believe they have such a fellowship. But that is not exactly the case.” MW14*

The midwives hoped for an increasing awareness in the Somali born women’s networks that the ANC clinic was a resource for situations concerning violence exposure and for psychological support during or after such occasions.

*”…also this with ripple effect. When someone starts…and then it spreads…so we should not give up and think ‘no, this doesn’t work’. … And then we might get more who attend and they see that this was not so bad, instead they are allowed to talk about it. Without it being spread or that anyone uses it against them. And eventually more and more people might be coming.” MW1*

## Discussion

This interview study with Swedish ANC midwives working with Somali born women conveyed a wish to provide individualized care, beyond culture or ethnic backgrounds. Almost all informants broached questions about violence exposure with Somali born women, but they seldom encountered ongoing violence. Limited communication and divergent life situations contributed to barriers in determining violence towards Somali born women.

The specific contributions midwifery can make to both preventive and supportive care are highlighted in the framework for quality maternal and newborn care (QMNC) [38]. The development of the framework is based on midwifery lead interventions associated with efficient use of resources with positive impact on health outcomes and on women’s perceptions. Women generally wanted “caring health care professionals who combine clinical knowledge and skills with interpersonal and cultural competence” [38]. Although not particularly focusing on violence; this framework identifies components of importance for women during the childbearing period of use to mirror findings in this study.

The midwives in our study stated that it should not be presupposed a Somali born woman knows her Swedish lawful rights or what kind of official support she has access to in a new society. Consequently, midwives took on ‘bridge-functions’, positioned in the gap between the Somali woman and the community. The midwives seldom associated Somali born women with high levels of violence exposure, despite several risk factors, such as foreign-born [15,16] and with origins in a low-income war-affected country [17,18]. However, even if no ongoing violence was detected, they could reach out with information and initiate reflections on violence and rights. Language barriers, different norm systems, fear from the women’s side of social risks, and other forums for support might contribute to non-disclosure of violence [39,40]. Combined with remaining adaptations related to pre-migration situations of war and insufficient societal support systems, risks for non-disclosure might increase [37]. However, a spontaneous association was made by several midwives between the ‘normal’ or ‘low’ findings of violence exposure and the strength and independence they perceived Somali born women often possessed. This is noteworthy, since it challenges stereotypic pictures of vulnerable refugee victims. Nor did the midwives perceive higher need and/or use of support for mental distress, rather the opposite. Alternative strategies for handling traumatic events were mentioned but the midwives also perceived that mental distress might be something the Somali born women were not used to sharing. Increased risks of unmet needs related to mental distress among migrants have been described [41], as have other conceptualizations of mental health and alternative treatments for mental illness in the Somali community [42]. Further research is needed to target potential needs adequately and to embrace alternative coping strategies among Somali women in these areas.

Shared language was an identified key issue when approaching violence with the Somali born woman. Mutual communication is craved by women in care encounters [38] and earlier research stress the importance of access to professional interpreters in care encounters between different languages [29,43]. However, despite routine provision of professional interpretation services, informants in our study sensed that communication barriers still remained. Fear of gossip, rendering less openness on the woman’s side, could be reduced to some extent with a telephone interpreter, but still a risk of misunderstanding and loss of nuance remained, leading to loss of control in the communication. This in turn risked reducing conversations about violence. Nevertheless, midwives provided evidence of ‘interpersonal competence’ [38] through using the divergent languages positively: sharing some Somali words reduced initial distances and could be a starting point for a deeper relationship. A trustful relationship has been identified in the recent framework for quality maternal and newborn health as an important factor for childbearing women [38]. This is confirmed in our study regarding communication about violence with the Somali born woman. Sometimes repeated appointments, or even pregnancies, were required, before violence was disclosed. Thus, our findings highlighted the importance of continuity of care [44], specifically in relation to immigrant women, alongside networking with other health care professionals and immigrant communities.

The midwives’ desire for individualized and nuanced approaches in their encounters with patients was a prominent finding. Knowledge about the women’s communities and provision of care suitable for the individual woman is identified as an important factor for providing quality maternal and newborn care [38]. It is furthermore argued that women in situations of vulnerability can gain increased mental, physical, and social health if the care offered by the health care system is based on individualized person- or patient-centered principles [45,46]. Thus, the person-centered approach identified among midwives in this study can be beneficial when approaching possible violence exposure towards women from a minority group. However, social distances [47] were depicted in the midwives’ reflections on different norm systems and they highlighted presumed divergent views on violence, family roles, and mental distress between the midwife and the Somali born women. The knowledge about how these issues were defined and handled by the Somali born woman and her networks were fragmentary. In the gap of social distances, a risk of constructing simplified explanations for behaviors on group-level is embedded [40,47]. Group-level perspectives should not be excluded in care encounters, for example, the risk factors for violence exposure can include groups of patients carrying certain commonalities. Negation of group-level factors in an unreflective quest for individuality may hence result in negative health outcomes. However, if group-level explanations have too much emphasis, a risk of simplification and stereotyping might render negative health outcomes. Therefore an effective person-centered approach needs to be grounded in both professional knowledge and knowledge about both one’s own and others’ values alongside interpersonal communication skills [38,48]. This knowledge is for a midwife suggested to consist of theoretical, experience based, and intuitive knowledge [49]. In our study, experience based and intuitive knowledge provided the basis for the midwives, but a deeper theoretical foundation and employers’ support to improve skills in intercultural communication was asked for. Furthermore, local facility based recommendations regarding violence questions did not sufficiently meet the needs they encountered with Somali born women due to their backgrounds in a war zone. Thus, midwives described different strategies and/or uncertainty in their encounters with Somali born women.

The unique role of the ANC midwife, repeatedly meeting women in reproductive age, offers a window of opportunity for reaching out to marginalized women in society; but requires time and flexibility. Furthermore, if potential hidden needs are to be identified and simplified pictures on group level to be avoided, midwives need to be provided with a foundation of culturally relevant knowledge and guidelines targeting a diverse population within a supportive organizational system.

### Methodological considerations and limitations

Throughout the study process measures were taken to achieve trustworthiness [50]. Credibility was addressed through a varied selection of ANC clinics in both small cities and larger metropolitan areas, through not interviewing more than two informants from the same clinic, and by all authors being involved in discussing data throughout the collection, analysis, and writing. One limitation might be that only midwives with extensive experience of working with Somali born women were selected, thus, the findings do not mirror perceptions of ANC midwives in Sweden in general. However, the purpose in qualitative research is not to deliver generalized facts, rather to contribute with depth and nuances, which was obtained in the present study. To ensure consistency, the same investigator conducted all interviews and continuous field notes provided the possibility to check dependability throughout the study periods. In qualitative research, the researcher is the main tool when gathering and analyzing data. Thus, the researchers’ own pre-understanding might affect conformability. The main investigator being also a midwife might have influenced the amount, content, and interpretation of the data. However, with the interviewer and interviewee being on equal terms as professionals meant the informants might be more prone to freely share experiences and reflections during the interviews. The choice of a well-described, structured analytic method, rich descriptions of findings and illustrating quotes [35] addressed the issue of transferability.

## Conclusions

Focus on the individual woman, supported by skills in inter-cultural communication increases the possibilities of overcoming social distances in the care encounter. This enhances midwives’ ability to identify Somali born woman’s own resources and needs regarding violence disclosure and support. Although routine use of professional interpretation is implemented, it might not fully provide nuances and social safety needed for violence disclosure. Thus, patience and trusting relationships are fundamental keys in work with violence among Somali born women. Through placing the midwifery work and the woman in a larger societal context, the midwife can act as a bridge, and contribute to increased awareness of rights and support for Somali-born women in a new society.
